# Material matters: a framework for integrating surface properties into built environment microbiome research

**DOI:** 10.1128/aem.02036-25

**Published:** 2026-02-10

**Authors:** Kobi Talma, Joana Sipe, Nathan Bossa, William Stiffler, Evan Hankinson, Claudia Gunsch, Mark Wiesner

**Affiliations:** 1 Department of Civil and Environmental Engineering, Duke University 3065, Durham, North Carolina, USA; 2 School of Integrated Engineering, Ira A Fulton Schools of Engineering, Arizona State University, Tempe, Arizona, USA; 3 School of Sustainable Engineering and the Built Environment, Ira A Fulton Schools of Engineering, Arizona State University, Tempe, Arizona, USA; 4 School for Engineering of Matter, Transport and Energy, Ira A Fulton Schools of Engineering, Arizona State University, Tempe, Arizona, USA; 5 Biodesign Center for Sustainable Macromolecular Materials and Manufacturing, Biodesign Institute, Arizona State University, Tempe, Arizona, USA; The Pennsylvania State University, University Park, Pennsylvania, USA

**Keywords:** microbiome of the built environment (MoBE), material-microbe interactions, surface properties, metadata reporting

## Abstract

The built environment (BE), where we spend the majority of our time, contains a variety of surfaces with distinct properties. Our understanding of how these surfaces shape the microbiome of the BE (MoBE) is underdeveloped and limits the ability to develop a bioinformed microbial management framework. Lab-scale studies have shown the impact of surface properties (roughness, wettability, porosity) on microbial communities, but studies sampling the BE microbiome have often overlooked this metadata. A keyword search of the literature found that only 31% of studies that sampled the indoor microbiome reported material information, which did not include any material characterization data. We have used the kitchen as a case study to illustrate the complexity of the microbial community and material surfaces that are present in the BE. We also describe how the use of BE spaces, such as cleaning, can impact both the materials and microbial community. We propose an interdisciplinary approach to studying the MoBE, incorporating techniques from material characterization into environmental microbiological sampling to elucidate the role of materials and their surface properties on the MoBE. Utilizing this interdisciplinary approach, a bioinformed framework can be developed for managing healthy MoBEs—one that improves occupant health by incorporating material science into microbial risk assessment and design strategies.

## INTRODUCTION

Built environments (BE) are composed of various materials with diverse chemistry, properties, and textures. When designing a home, many homeowners or interior designers consider the aesthetics and materials used to create each room and component; however, their effect on the microbiome is poorly described. Dai et al. describe how an engineering approach is needed to understand the factors for the BE exposomes and their influence on human well-being (1). This is because humans spend more than 90% of their time indoors, and most of this time is in residential locations (2). Although mechanisms of transmission are well understood, more can be learned about how BE design influences the proliferation or transmission of infectious microorganisms (3). The current framework of BE microbial management is ineffective, and the development of a bioinformed framework shows great promise for creating healthy microbiomes of the BE (MoBEs). Understanding how surfaces impact bacteria is key for this solution to gain more traction.

**Fig 1:**
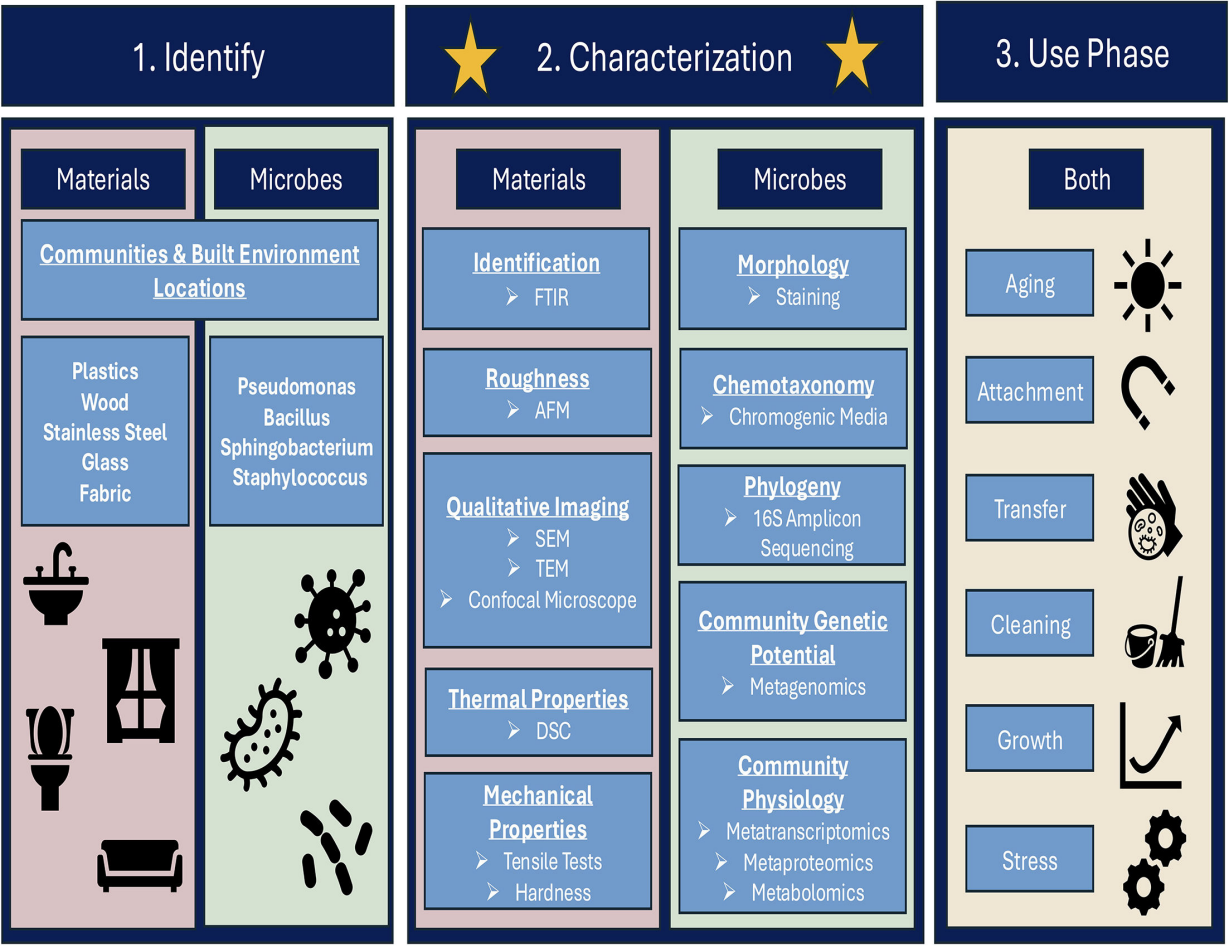
Flowchart for assessing material and microbial characterization.

In general, there is a gap in understanding how surfaces impact the microbial communities that exist in the BE, and especially how these surfaces can be engineered to minimize the growth of microbes that cause illness or promote the colonization of microbes associated with health. Additionally, there is a lack of an integrated framework that incorporates building design, material use, environmental factors, and the microbiome to assess potential health risks in the BE. This review helps develop a framework for characterizing material properties in BE microbial community studies to better understand the influence of materials on the microbiome and, ultimately, microbial risk indoors. This can help further the studies investigating engineered surfaces and provide insight into how to deliberately engineer MoBE. A proposed workflow for designing experiments testing the microbiome community and materials they are in contact with is shown in Fig. 1.

Microbiome-surface interactions have been studied in the context of microplastics (4, 5), bioelectrochemical systems (6, 7), dentistry (8), and biological wastewater treatment (9). These studies showed that material properties, such as surface hydrophobicity, roughness, and charge, can influence microbial diversity and community composition. However, microbiome-surface interactions in the BE have not been extensively studied. This review summarizes knowledge on microbial interactions with surfaces, applying this knowledge to kitchen surfaces in order to focus on the characterization process and a subset of materials present in the room. The kitchen was chosen due to diverse microbial communities found in the kitchen (10) and the exchange of microbes to humans dermally, via inhalation, or ingestion (11). In addition, surfaces in the kitchen have the ability to harbor microbial communities that may be sources of pathogenic microbes within the BE. The importance of surface coatings and cleaning of surfaces to microbial adhesion and transfer will also be discussed, as transmission and hygiene are essential in household kitchen environments (12).

## INDOOR MICROBIOME STUDIES AND MATERIALS

A keyword search was carried out across two academic search engines (Science Direct and Clarivate Web of Science). The following keywords were selected for their relevance to the topic and to ensure a representative number of results were obtained:

built environment AND indoor AND surface AND bacteria AND sampling AND material NOT review

The results of this search were further filtered by considering only literature published in 2024 or before and considering only literature published in English, resulting in a total of 443 studies. The following information was recorded for each result of the keyword search:

1. Was the indoor microbiome sampled? (Y/N)
2. Were human pathogens found/reported? (Y/N)
3. Was the location of sampling within the indoor microbiome reported? (Y/N)
4. Was the material sampled reported? (Y/N)

Of the 443 studies sampled, 124 examined the indoor microbiome, indicating that our keyword search selection was broad. The following analysis was performed only with research sampling the indoor microbiome. The indoor microbiome was sampled predominantly from the air (49%), followed by surfaces (34%), dust (7%), water (5%), and other materials (e.g., food, debris, waste).

**Fig 2:**
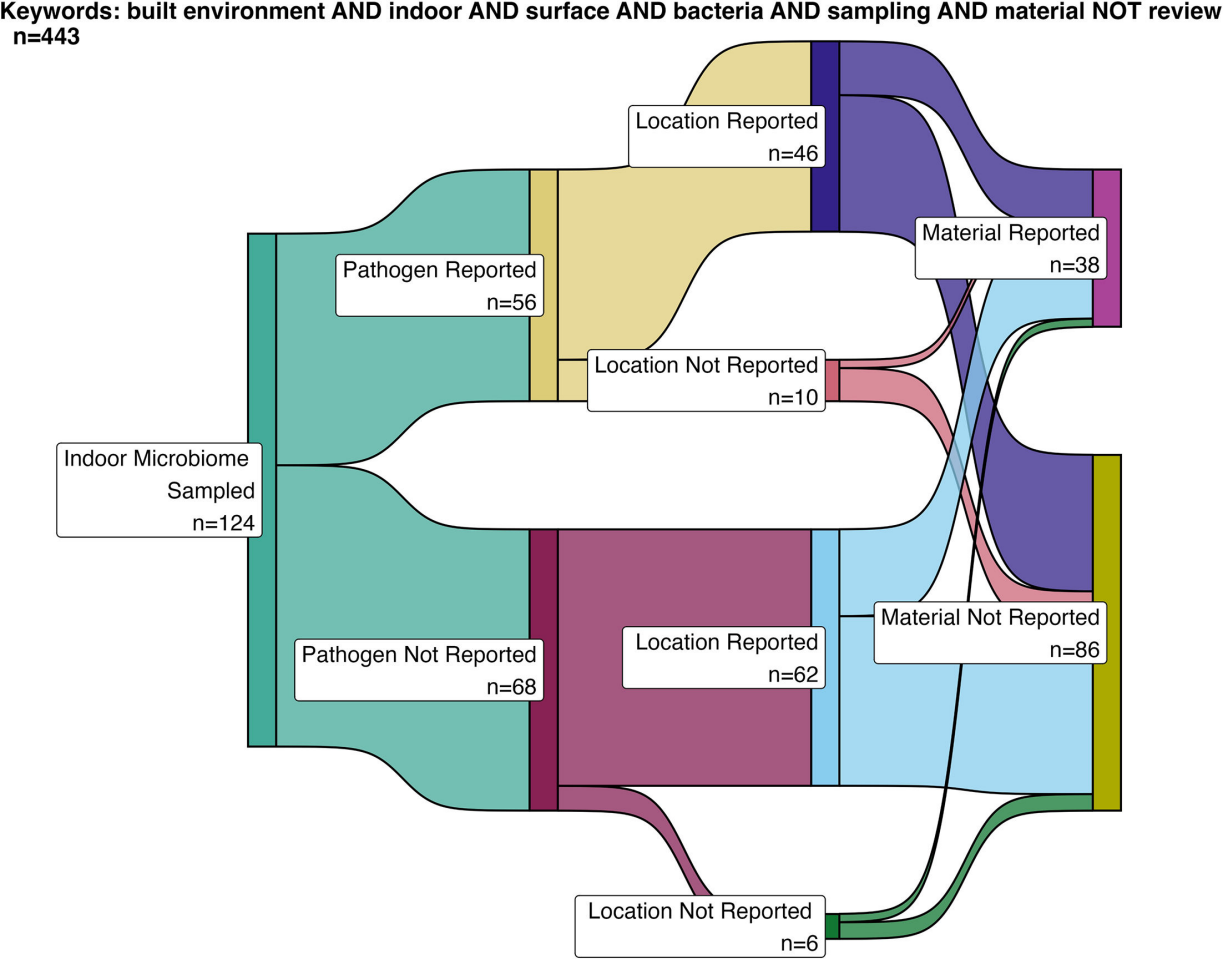
Sankey diagram illustrating the number of studies that sampled the indoor microbiome and the metadata reported. From left to right: pathogen reported (Y/N); location reported (Y/N); and material reported (Y/N).

Figure 2 shows that the number of indoor microbiome studies that reported pathogens was similar to the number that did not report pathogens, with 52 and 68 studies, respectively. Bacterial pathogens were the most frequently reported (53%), followed by fungi (43%), protozoa (2%), viruses (1%), and nematodes (1%).

The location of sampling was reported in 108 studies, with 46 of those studies also reporting pathogens, while 62 did not report pathogens. Indoor microbiome sampling was most frequently conducted in residential buildings (26%), followed by public buildings (20%), other settings (19%), laboratories and offices (10% each), hospitals (8%), churches (4%), and hotels (2%). Only 25 entries were more specific in the location, which for residential spaces included bedrooms, kitchens, bathrooms, and living rooms. For public and institutional buildings, spaces included classrooms, activity rooms, and offices. Shared public touch surfaces included ATM keypads, doormats, and gym facilities, and other locations included HVAC systems, boots, washing machines, and sludge-handling facilities.

Among the different articles, only 38 (31%) provide material information. Plastic surfaces were the most frequently studied, representing 20% of all materials, followed by wood (15%). Metal and stone each accounted for 12% and 12%, respectively. Less commonly sampled materials included carpet and paint (5% and 6%, respectively), and concrete, glass, gypsum, and plaster (each 4%). Tile was the least represented material at 2%. Other materials, such as meat and soil, represented 12% of the materials reported. Among the 124 studies returned by the keyword search, only the material name is listed, and none of them report characterization data. This brief overview of studies highlights the lack of a clear description of the materials and material characteristics within environmental studies of the indoor microbiome. To address this gap, this review draws upon lab-based research—particularly from fields where materials engineering plays a central role—to provide insight into how material properties influence microbial behavior.

## PROPERTIES INFLUENCING BACTERIAL ATTACHMENT AND BIOFILM FORMATION

Bacteria can induce physicochemical changes in the microenvironment near surfaces, affecting factors such as pH (13) and releasing organic molecules (14–16). Antibacterial materials incorporating coatings or biocide-encapsulated agents that release chemical agents are not discussed in this section.

### Contact point, type of binding of bacteria to materials

The initial stage of bacterial adhesion, known as reversible adhesion, involves the early attraction of planktonic bacteria to a substrate surface. During this phase, bacteria accumulate on the surface through passive and/or active movement, yet they still exhibit random movement within a medium (Brownian motion). In this early stage, attractive (e.g., van der Waals) and repulsive (e.g., electrostatic) physicochemical forces partially mediate the interactions between the bacterial and substrate surfaces. The chemical properties of both surfaces and the surrounding liquid environment (17) influence these forces. To some extent, these forces have been modeled using the classic and extended DLVO theory (18).

When attractive forces exceed repulsive forces, bacterial adhesion proceeds to a more stable phase. In irreversible adhesion, cells attach firmly to the substrate, reinforcing this attachment through molecular and cellular interactions, as well as through the production of specialized adhesion molecules.

Understanding these adhesion processes has led many studies to investigate how material properties influence bacterial attachment. Research typically focuses on materials designed for specific applications, including dental implants (19), medical materials (textiles, plastics, etc.) (20), contact lenses (21), membrane filtration (22), and recently, the biodegradation of micro/nano plastics (23). In the following sections, the effect of surface roughness, surface wettability, and porosity will be detailed separately, with some focus on the effect of surface charge and free energy when relevant.

### Surface roughness

Surface roughness is a quantitative measurement of a material’s surface topography, which defines the surface area available for bacterial contact. The typical roughness values of kitchen materials are described in “Properties of typical kitchen materials,” below. The impact of roughness on bacterial adhesion has been studied at various scales, from nanometric to micrometric. At the nanometric scale, roughness influences the number of favorable attachment sites, while at the micrometric scale, roughness could provide a protected environment for bacteria, with favorable conditions such as enhanced humidity and food.

A recent paper plotted the impact of roughness as a function of bacterial adhesion, which was extracted from 16 studies, revealing that at the nanoscale, bacterial adhesion increases as surfaces become smoother, and the plotted results suggest a threshold at 6 nm (24). Furthermore, adhesion increases as surfaces become rougher at the microscale, showing maximum adhesion of cells when microscale roughness is present. The effect of roughness at the nanoscale is debatable, and the inhibition of adhesion by the nanoscale roughness is not always trivial. Some bias might be found in how the nanoscale roughness is generated or created, as well as the wettability of the material, or simply with the goal of studies that aim to create nanostructured materials with antimicrobial properties. Mu et al. tested bacterial attachment to 14 hydrophobic quartz materials with nano-roughness scales spanning from ∼2 to ∼390 nm using Gram-negative *Salmonella typhimurium* LT2 and *Escherichia coli* O157:H7, as well as Gram-positive *Listeria innocua* (25). At low roughness (RMS < 10 nm), surfaces showed isolated microcolonies with few adherent bacteria and low areal density, surrounded by extracellular polymeric substances. At intermediate roughness (RMS 10–40 nm), adherent bacteria increased linearly as a function of roughness, forming loosely connected monolayers and appeared more deformed, suggesting stronger surface attraction. At high roughness (RMS > 45 nm), bacterial density was very low, with mostly single, isolated cells and occasional small aggregates. This was caused by the increase in roughness, leading to an increase in the water contact angle and induced air bubble surface accumulation that reduced the effective substrate area for bacteria.

At the microscale, increased roughness provides more protected spaces for bacteria, reducing their mobility and making them harder to remove during cleaning. Bohinc et al. prepared glass surfaces with five different roughness values (0.07, 0.58, 0.99, 2.5, and 5.8 μm) and observed that the rate of *Escherichia coli*, *Staphylococcus aureus*, and *Pseudomonas aeruginosa* adhesion increased with increasing surface roughness (26). Some studies have proposed the idea of a threshold arithmetic average roughness (Ra) of 0.2 μm, where up to this threshold, adhesion is largely improved. For example, an *in vitro* study claimed that threshold values for the adhesion of *Streptococcus mutans* and *Streptococcus sobrinus* to composite resin surfaces were estimated between 0.15 and 0.35 μm (8). Considering this, the European Hygienic Engineering and Design Group, the American Meat Institute Equipment Design Task Force, and 3-A Sanitary Standards, Inc. recommended surface roughness of less than 0.8 μm for kitchen countertops (27). An increase in micrometric roughness not only increases bacteria adhesion but also increases surface soiling and reduces surface cleanability. Roughness features that exceed bacterial dimensions can permit cells to settle within surface depressions, thereby increasing the flow velocities required for their detachment—levels that might not be achievable with conventional cleaning utensils (28). Embedding probiotic spores into surfaces, including fabrics (29) and 3D printed materials (30), has shown promise for incorporating beneficial microbial communities into materials. The impact on material cleanability is uncertain, and balancing the benefits of the probiotic with the potential for pathogen accumulation is an opportunity for future study.

A key limitation in most studies examining surface roughness is that analyses are performed at one scale. Researchers typically either conduct AFM analysis, which measures nanometric surface variations within frame sizes of 10 to 50 microns, or use optical interferometric profilometers, which are suitable for submicron and micrometric analyses, depending on the optical magnification. A multi-scale approach to measuring roughness should be considered in future studies.

### Surface wettability

When examining the impact of chemical structure, hydrophilicity or hydrophobicity, surface charge, and the surface free energy of material surfaces on bacterial adhesion, it is important to highlight the strong interdependence of these factors. The chemical structure and functional groups present in both natural and synthetic polymers influence their hydrophilicity and surface charge. Furthermore, hydrophilicity is closely linked to the surface free energy of the surfaces, as more hydrophilic materials exhibit higher surface free energy, and a combination of surface chemistry and surface roughness influences surface wettability.

The water contact angle (WCA) on rough surfaces differs from the intrinsic WCA on smooth surfaces. Superhydrophobic surfaces (WCA > 150°) are formed using nanostructures and are explained by the Cassie–Baxter and Wenzel models. The Cassie–Baxter model involves droplets resting on microstructures with air underneath, while the Wenzel model involves droplets penetrating the surface texture, leading to stronger adhesion (31, 32). Beaussart et al. studied *Lactobacillus plantarum* using bacteria attached to AFM tips (33). The hydrophobic layer showed adhesion forces ranging from 250 to 2,500 pN, with multiple sequential peaks and extended rupture lengths. In contrast, on hydrophilic substrates, a single, well-defined force peak of approximately 200 pN was recorded.

It is also evident that the probability of bacterial attachment may be increased on hydrophobic surfaces. When this occurs, hydrophobic surfaces reduce the near-wall velocity of bacteria through collisions and slightly increase the collision duration (34). Interestingly, the effect of surface wettability on the rigidity of bacterial membranes has been quantified, showing that hydrophobic surfaces render the bacterial outer membrane relatively “soft,” while hydrophilic surfaces maximize contact area with the substrate (35). Furthermore, on hydrophobic surfaces, bacteria are better able to withstand higher applied lateral forces. The number of dislodged bacteria is lower, and the dislodgement forces are correlated with initial adhesion forces in the attachment process, as both inversely follow surface energy trends (36).

The attachment of *Staphylococcus epidermidis* to tunable poly(N-isopropylacrylamide) showed a weak correlation to the contact angle, while *Cobetia marina* presented a stronger correlation (37). Li et al. studied the adhesion strength of eight bacterial strains on glass and iron surfaces with varying contact angles (38). They found that adhesion was significantly correlated with the glass surface water contact angle. The authors concluded that the correlation of adhesion with surface energy (based on three liquid contact angles) was the most reliable predicting parameter and that there is a correlation between the surface energy of substrates and the interaction energy of bacteria to the substrates. The number of cell colonies attached to the surfaces decreased with decreasing surface energy or with increasing total interaction energy (Δ*E*^TOT^) (39).

Other models confirm this finding with the adhesion of *Pseudomonas putida* on hematite, found to be greater than on quartz due to the physical-chemical properties and surface hydrophobicity (40). Despite great success in using DLVO theory to predict bacteria attachment, Bos et al. state that “a physico-chemical approach will most likely never be able to fully explain all aspects of microbial adhesion to surfaces, including interspecies binding (41).” Incorporating microbial transcriptomics or proteomics could provide the required information to get a full understanding of adhesion to surfaces. Transcriptomics can provide information on gene regulation (42) and can elucidate biological details pertinent to bacterial adhesion. Similarly, proteomics measures and identifies the proteins present (43), which can influence the formation of biofilms (44).

### Porosity

The influence of textile water adsorption capacity and drying kinetics has been demonstrated. Significant differences in drying rates were observed among fabrics, with *Salmonella* growth being more pronounced in damp cloths. Pathogens exhibited reduced survival on hanging cloths, which dry faster compared to those left near the sink. Similarly, *Campylobacter*, *Salmonella,* and kitchen-associated bacteria were studied in three types of sponges and one type of brush. The lowest bacterial numbers were found in brushes, where a rapid die-off of all types of bacteria was observed (45). The effect of humidity on adsorption was also evident, with wood preparation tables having a higher prevalence of coliform and *Enterococcus* spp. compared to inorganic kitchen surfaces. The nature of wood, which is porous, might allow penetration of juices from foods and bacteria, hence preventing their removal during cleaning and favoring their colonization (46). As described in later sections, sponges are a major bacterial carrier; however, we did not find any study on the effect of sponge porosity, pore size, or water retention. Nonetheless, it was reported that washing of sponges contaminated with food did not reduce the bacterial load significantly (47), indicating that the porous properties of sponges may offer a decent refuge for bacteria even during cleaning processes.

In the kitchen, wood, textiles, sponges, and ceramics are among the materials that exhibit porosity. In medical applications, porous materials have a higher level of infection compared to dense materials (48, 49). Similarly to microroughness, bacteria live on porous surfaces whose pore size corresponds to the size of the bacterial cell, as the porosity and bacteria-adapted pore size increase the contact between the bacteria themselves and the surface, which in turn increases the adhesion potential and promotes growth (50). However, the porous media need to be large enough to let bacteria in, but more precisely, the pore volume needs to be large enough to let bacteria enter. For example, porous materials such as hydroxyapatite and biphasic calcium phosphate ceramic materials do not have pores sufficiently large to allow the internalization of *Staphylococci* (51). Porosity not only allows for the housing and protection of microbiomes from cleaning but also aids in their transport to other surfaces or interaction with various contaminants or materials of interest. Sealing of countertops, which has typically been advertised to prevent the absorption of liquids, could be implemented to limit microbial colonization. However, the impact of sealant coatings on other surface properties must be considered, as these properties can also influence bacterial attachment and colonization.

### Chemical and molecular level responses

The specific nature of bacterial surfaces cannot be neglected, and other mechanisms allow bacteria to adhere despite repulsive hydrodynamic forces (52). Bacteria use a variety of mechanosensory and chemical signaling mechanisms to detect and respond to surface contact. Reviews by Kimkes et al. and Kreve et al. outline the signals bacteria perceive upon attachment (53, 54). Specialized appendages such as flagella and pili mediate specific binding to chemical groups on surfaces (55), and flagella can enhance initial attachment by helping cells overcome energy barriers and adhere more effectively to materials such as plastics (56). These appendages also carry receptors that sense physical cues like roughness and hydrophobicity, as well as chemical signals, including nutrients or host-derived molecules (57). Upon initial attachment, bacteria activate signaling pathways—often via two-component systems—that regulate gene expression and metabolism in response to surface contact (53). Production of microbial exopolysaccharides can aid in attachment and play a role in biofilm communities (58). Highly specific coaggregation interactions further stabilize attachment, requiring adhesins on one cell to recognize receptors on another (59). Additional cues such as flagella morphology (60), metabolic shifts upon contact (61), or shear-dependent activation of adhesion molecules (62) also shape attachment behavior.

## INTERACTION BETWEEN KITCHEN SURFACES AND THE KITCHEN MICROBIOME

**Fig 3:**
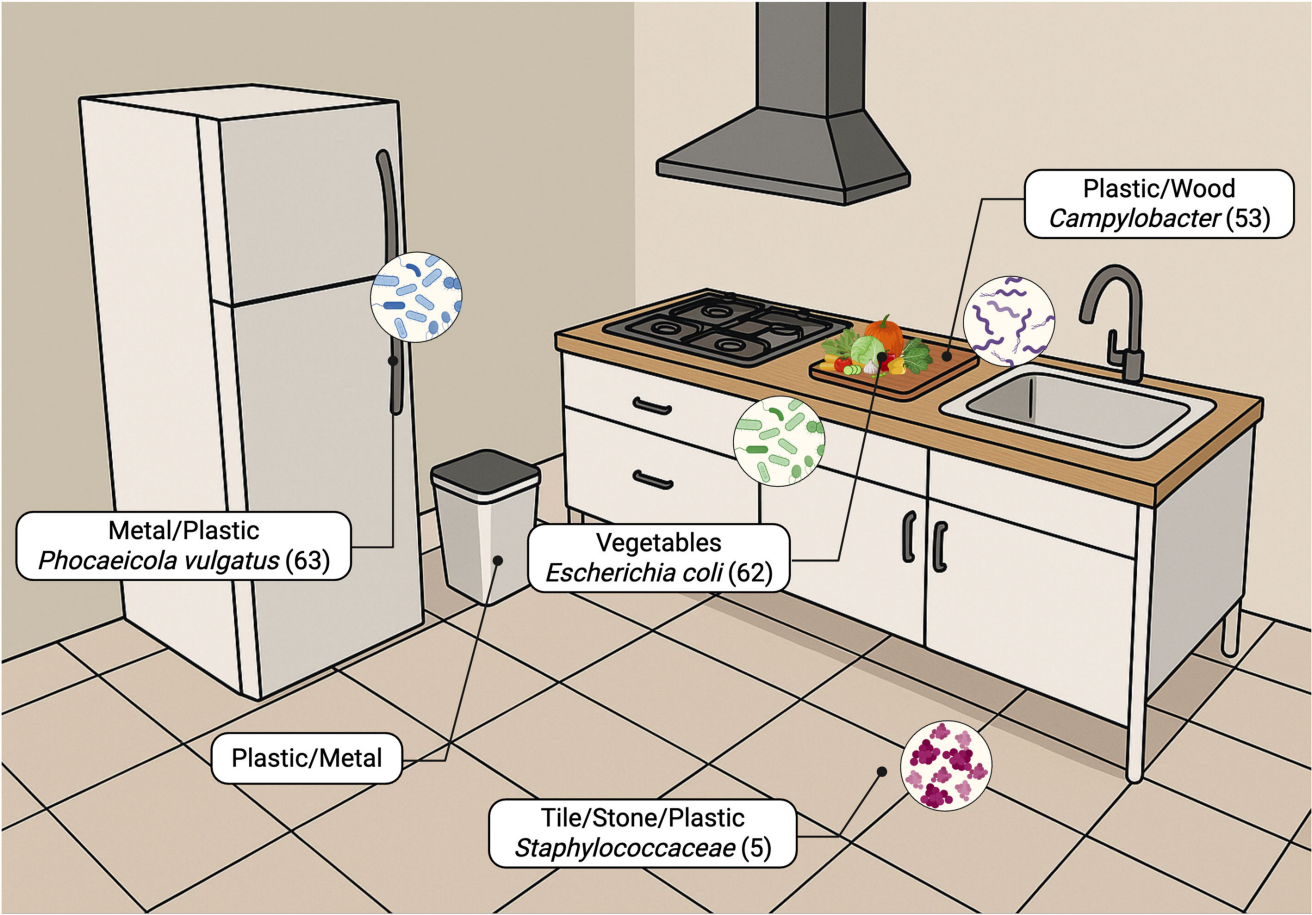
Microbial hotspots in the kitchen and common material types.

We present the kitchen as an example of a BE that contains various surface materials and harbors complex microbial communities to emphasize the relevance of material properties in MoBE research (Fig. 3). The connections between materials and microbes in the BE presented below are relevant in other settings, particularly those in which pathogen exposure can result in negative health outcomes.

The kitchen is a well-studied BE microbiome due to its diverse materials, fluctuating temperature and humidity, and strong influence from social behaviors, and because material types can vary across socioeconomic contexts—for example, wealthy households historically used antibacterial silver cutlery—while surfaces in low-income homes may age more, increasing roughness, promoting bacterial attachment, and reducing cleanability. Together, these factors make kitchens in residential, commercial, institutional, industrial, and hospital settings critical hubs for microbial exchange and a central focus for microbiome research.

When human contact is controlled for, the MoBE is principally dictated by location and geography (63). Even without direct contact, 30% of this microbiome is shared with the human skin microbiome, indicating that the inhabitants play a major role in shaping this ecosystem. The authors concluded that in dry and nutrient-poor areas like this, material played little role in the microbiome. The most nutrient-rich areas of the BE, however, have the potential to support ecosystems.

The kitchen is an area of high interaction between human and microbial members of the BE. To combat this, we modify our environment by sealing off segments of the BE to be cooled to temperatures that efficiently minimize the metabolic activity of microbes (refrigerators) or cease activity altogether (freezers), with exceptions (64). However, when at room temperature and on kitchen surfaces, foodstuffs not only act as nutrient sources, but also as microbial sources. Different categories of foodstuffs are associated with unique microbial communities, which have been shown to differ based on storage, manufacturing conditions, and origin (65, 66). It is suggested that the microbiome of our fruit and vegetable intake can be incorporated into our gut biome (67). More recently, studies have probed how the amalgam of ambient, human-associated, and food-associated microbes interacts in the rich and complex kitchen ecosystem, as described in the following section.

### Common bacteria

A comprehensive analysis of five different surface samples in 74 households across five European countries identified 31 bacterial phyla: Proteobacteria, Firmicutes, Bacteroidota, and Actinobacteria being the most prevalent (68). This study succeeded in elucidating a core kitchen microbiome shared across countries and cultures. Another exhaustive study of over 80 kitchen surfaces across four households recapitulated the major phyla found above and added increased resolution into the extent of bacterial colonization on kitchen surfaces and the role of human beings in shaping these microbiomes (11). This study showed that taxa exhibited distinct distribution patterns among the kitchen environment, with the most diverse assemblages occupying areas cleaned most infrequently, whereas often-cleaned surfaces like sinks were dominated by Gram-negative biofilm-formers. Carstens et al. examined 10 households in Houston, TX—likewise sampling from different regions of the kitchen environment (69). This study corroborated cleaning-diversity correlations of the prior two; it focused predominantly on specific taxa, as opposed to phyla, finding *Pseudomonas* to dominate sinks and counters. The presence of Gram-negative bacteria on often-cleaned surfaces is of particular interest as these species are more likely to be resistant (70), in addition to being more likely to acquire antibiotic resistance genes (ARGs) through horizontal gene transfer (71).

Special attention is often paid to pathogen presence in the kitchen environment. Pathogenic species common to the kitchen environment, *Escherichia coli, Staphylococcus aureus,* and *Klebsiella pneumoniae,* are all highly persistent and resilient species capable of quickly establishing populations when environmental conditions are favorable. However, it is slower-growing species, such as *Campylobacter jejuni* and *Salmonella enterica,* that are among the most common bacterial causes of foodborne illness in the U.S. (72) and Europe (73). This could reflect infrequent cleaning as a major driver of foodborne illness or could indicate that these microbes have persisted in an adjacent, infrequently cleaned environment. Kotay et al. (74) demonstrated that microbes in the sink p-trap can spread up to 30 inches from the tailpipe via droplet-mediated dispersion (74).

Although human behaviors appear to have an outsized effect on the microbial makeup of the kitchen environment, the human biome’s contribution is varied. A study comparing 10 Korean households’ refrigerator door handles to data from the Human Microbiome Project found only 15.6% of species from handles were shared with the human skin biome and 4.9% with the human gut biome (75). Conversely, Flores et al. tracked indicator species distinct to specific sources and found that the most important source of microbes was human skin compared to faucet water and produce (11). The discrepancy in findings may stem from issues in the sampling process. ISO 18593:2018*—Microbiology of the food chain—Horizontal methods for surface sampling* is the main standardized procedure for surface sampling in built environments using swabs, contact plates, and sponges, aiming to improve reproducibility in microbial monitoring (76). However, material properties significantly influence sampling efficiency (77), with recovery rates ranging from ~30% to 70% on non-porous hydrophilic surfaces like stainless steel to less than 10% on porous materials, such as unsealed wood, due to absorption and surface roughness (76, 78). Hydrophobic surfaces, including many plastics, also yield lower recoveries (10%–30%) without surfactant-containing buffers because of poor wetting and microbial adhesion strength (76, 79).

Additionally, bacterial characteristics critically affect recovery. Gram-positive bacteria (e.g., *Staphylococcus aureus*) are generally recovered at higher rates compared to Gram-negative bacteria (e.g., *Escherichia coli*), a trend attributed to differences in cell wall robustness and desiccation tolerance (76). Spore-forming bacteria (e.g., *Bacillus* spp.) exhibit moderate recovery (e.g., ~48% on stainless steel and ceramic surfaces using sponge wipes), although they adhere strongly to both smooth and rough surfaces (79, 80). Biofilm-forming bacteria typically show very low recovery (<1%–10%) due to the protective nature of their extracellular matrix. For instance, in a study on *Listeria monocytogenes* biofilms, sonicating swabs recovered significantly more cells and left less residual biomass (median ~1.1%) compared to conventional cotton swabs (median ~70.4%) (81). Overall, while ISO 18593:2018 ensures procedural standardization, quantitative interpretation requires careful consideration of both surface material properties and bacterial physiological traits to avoid underestimating microbial contamination in environmental assessments (82).

Regardless of the myriad sampling discrepancies affecting conclusions on kitchen microbiome sources, it is undeniable that the kitchen microbiome is distinct from its inhabitants, being comprised of microbes from external sources, such as the outdoor environment and foodstuffs, in addition to the human microbiome. However, there appears to be a convergence on a core kitchen microbiome across countries and cultures. Increased reporting of material surfaces alongside sampling efforts could allow for more generalized conclusions that may be applied to better inform kitchen design.

### Material surfaces in the kitchen

Studies on the microbiome within the residential home have focused either on locations and surfaces that were expected to harbor distinct communities or “high-touch” areas (83, 84). Kitchen surfaces include various plastics, wood, steel, glass, stone, and food surfaces. Most of the polymer surfaces were found on appliance surfaces, plastic cutlery, kitchen sponges, kitchen accessories (such as cutting boards), and food packaging. Some of the polymers of interest include various polyethylenes, rubbers, and polyacrylics (85). Wooden surfaces are found on dining tables, preparation tables, and cutting boards (46, 86). Sponges are of particular interest for microbes on kitchen surfaces, as they can transfer microbes to other surfaces. Rossi et al. found that contaminated sponges could transfer a large number of microorganisms to both stainless steel and polyethylene surfaces (87).

### Properties of typical kitchen materials

There is a wide range of reported surface properties of kitchen materials due to the variability of materials used. A few of the important surface properties that impact microbial attachment and the microbiome are discussed in a previous section. Studies that take environmental samples generally have not collected data about material characteristics, as discussed in “Indoor microbiome studies and materials,” above, but food safety literature has explored the characteristics of common kitchen material surfaces and bacterial activity in laboratory settings. The most common materials studied in this context were polypropylene, granite, and stainless steel. Table 1 presents a summary of material characteristics reported in the literature for common kitchen materials.

**TABLE 1:** Material characteristics reported in studies of kitchen surfaces and microbial attachment^*a*^

Group	Material	Roughness—Ra (nm)	Water contact angle (°)	Bacteria	Source
Ceramics	Marble				
		22^*b*^^,*g*^	71.0 ± 1.0	*S. aureus*, *S. xylosus*	Azelmad et al. (88)
		8.9 ± 0.2^*c*^	76.0 ± 2.7	*L. monocytogenes* ATCC 15313	Teixeira et al. (89)
		8.5 ± 0.4^*d*^	66.3 ± 1.8	*L. monocytogenes* strains 1562, 994, 930, 925, 747, 923, 832, 812, 924, 1559	Silva et al. (90)
	Granite				
		9^*b*^^,*g*^	82.3 ± 0.6	*S. aureus*, *S. xylosus*	Azelmad et al. (88)
		43.9 ± 17.9^*c*^	49.2 ± 2.3	*L. monocytogenes* ATCC 15313	Teixeira et al. (89)
		13.1 ± 2.3^*d*^	57.4 ± 2.9	*L. monocytogenes* strains 1562, 994, 930, 925, 747, 923, 832, 812, 924, 1559	Silva et al. (90)
		32,400 ± 9,200; 24,900 ± 2,000^*e*^	53.4 ± 3.6	*S. Enteritidis* strains EMB, MUSC, PC, AL	Oliveira et al. (91)
	Ceramic				
		24.6 ± 4.0^*e*^	22.35 ± 1.80	*E. coli* ATCC 25922, *P. aeruginosa* ATCC 27583, *C. jejuni* 81-176	Zore et al. (92)
	Silestone				
	White	31.5 ± 1.5^*d*^	65.4 ± 1.9	*L. monocytogenes* strains 1562, 994, 930, 925, 747, 923, 832, 812, 924, 1559	Silva et al. (90)
	Beige	24.6 ± 6.4^*d*^	57.9 ± 3.0	*L. monocytogenes* strains 1562, 994, 930, 925, 747, 923, 832, 812, 924, 1559	Silva et al. (90)
Polymers	Polypropylene				
		16^*b*^^,*g*^	105.2 ± 0.5	*S. aureus*, *S. xylosus*	Azelmad et al. (88)
		6,200 ± 300; 200 ± 40^*e*^	87.8 ± 3.4	*S. Enteritidis* strains EMB, MUSC, PC, AL	Oliveira et al. (91)
	Kitchen bowl	14.1 ± 6.1^*c*^	109.6 ± 3.5	*L. monocytogenes* ATCC 15313	Teixeira et al. (89)
		4.8 ± 0.6^*d*^	97.1 ± 2.3	*L. monocytogenes* strains 1562, 994, 930, 925, 747, 923, 832, 812, 924, 1559	Silva et al. (90)
	Cutting board	17.5 ± 0.5^*c*^	95.5 ± 3.6	*L. monocytogenes* ATCC 15313	Teixeira et al. (89)
		8.5 ± 1.6^*d*^	89.3 ± 1.9	*L. monocytogenes strains* 1562, 994, 930, 925, 747, 923, 832, 812, 924, 1559	Silva et al. (90)
	Polyethylene				
		3.6 × 10^4^ ± 1.8 × 10^3^; 30,900 ± 8,900^*e*^	74.3 ± 8.3	*S. Enteritidis* strains EMB, MUSC, PC, AL	Oliveira et al. (91)
	Teflon (PFTE)				
		30.5 ± 3.0^*f*^	102.74 ± 1.50	*E. coli* ATCC 25922, *P. aeruginosa* ATCC 27583, *C. jejuni* 81-176	Zore et al. (92)
		384 ± 32^*f*^	101.93 ± 2.900	*P. aeruginosa* ATCC 27853, *S. aureus* ATCC 25923, *E. coli* ATCC 35218	Fink et al. (93)
	Polyethylene terephthalate (PET)				
		1,213 ± 55^*f*^	85.236 ± 2.753	*P. aeruginosa* ATCC 27853, *S. aureus* ATCC 25923, *E. coli* ATCC 35218	Fink et al. (93)
	Silicone				
		882 ± 212^*f*^	97.207 ± 0.748	*P. aeruginosa* ATCC 27853, *S. aureus* ATCC 25923, *E. coli* ATCC 35218	Fink et al. (93)
Metals	Stainless steel 304				
		3^*b*^^,*g*^	104.6 ± 0.9	*S. aureus*, *S. xylosus*	Azelmad et al. (88)
		81.6 ± 52.8^*c*^	81.3 ± 2.6	*L. monocytogenes* ATCC 15313	Teixeira et al. (89)
		30.9 ± 4.4^*d*^	90.4 *±* 2.9	*L. monocytogenes strains 1562, 994, 930, 925, 747, 923, 832, 812, 924, 1559*	Silva et al. (90)
	Stainless steel 316				
		6^*b*^^,*g*^	64.0 ± 2.0	*S. aureus*, *S. xylosus*	Azelmad et al. (88)
	Aluminum				
		229 ± 11^*f*^	62.328 ± 4.253	*P. aeruginosa* ATCC 27853, *S. aureus* ATCC 25923, *E. coli* ATCC 35218	Fink et al. (93)
Glass	Glass				
		1.3 ± 0.9^*c*^	49.7 ± 2.0	*L. monocytogenes* ATCC 15313	Teixeira et al. (89)
		1.6 ± 0.2^*d*^	69.9 ± 3.8	*L. monocytogenes* strains 1562, 994, 930, 925, 747, 923, 832, 812, 924, 1559	Silva et al. (90)
		990 ± 21^*f*^	45.311 *±* 8.608	*P. aeruginosa* ATCC 27853, *S. aureus* ATCC 25923, *E. coli* ATCC 35218	Fink et al. (93)

^*a*^ Superscript letters describe material characteristic measurement techniques.

^*b*^ Atomic force microscopy (8 μm × 8 μm area).

^*c*^ Atomic force microscopy (10 μm × 10 μm area).

^*d*^ Atomic force microscopy (2.5 μm × 2.5 μm area).

^*e*^ Non-contact laser stylus tracing.

^*f*^ Profilometry.

^*g*^ Values estimated from the figure.

Roughness measurements of common materials are reported at varying scales, with values in both the nanometer scale (88–90, 92) and micrometer scale (91, 93) found in the literature. The mode of measurement used influences the results of roughness in these studies, as described in “Surface roughness,” above. At the nanometer scale, metal materials have the highest roughness, and glass materials have the lowest roughness. However, at the micrometer scale, ceramic materials and polymeric materials have much greater roughness values than metal materials and glass materials.

Hydrophobicity is another frequently reported characteristic of kitchen materials experimentally evaluated for microbial activity. Polymeric kitchen surfaces, including polypropylene, polyethylene, and Teflon, are generally hydrophobic (88–93). Stainless steel was generally hydrophobic (89, 90), but different formulations of the material may play a role in the hydrophobicity of the surface (88). Materials commonly used for flat surfaces, such as granite, marble, and glass, vary in their hydrophobicity, which may be due to their origin, manufacturing, or finishing (88–92). Different formulations of UV-curable coatings for marble kitchen surfaces resulted in differing hydrophobicity, measured as water contact angle (94). Kitchen surfaces and their physicochemical properties can vary widely and have the potential to shape the kitchen microbiome, as a result, as described in “Properties influencing bacterial attachment and biofilm formation,” above.

### Cleaning

Cleaning is an integral part of behavior in kitchens, impacting the microbial communities on surfaces and the surfaces themselves. It must also be noted that surface characteristics, such as roughness or hydrophobicity, can also impact the effectiveness of disinfectants and other cleaning methods. High surface roughness of stainless steel surfaces resulted in limited cleaning effectiveness of minced meat and spinach spoils (95). Cleaning can result in physical changes to surfaces, through scrubbing and other similar techniques, and chemical changes to surfaces, from the use of disinfectants. A study evaluating the wear of ceramic tiles, to simulate kitchen working surfaces, investigated the impact of both wet and dry cleaning on surface roughness (96). They found that surfaces that were initially textured showed an insignificant increase in roughness after cleaning, while surfaces that were initially smooth exhibited a significant decrease in roughness after cleaning (96). The decrease in roughness of the initially smooth surface was less pronounced for samples cleaned with detergent, which acted as a lubricant, reducing mechanical stress on the surface (96). Chaturongkasumrit et al. found that the surface roughness of polyurethane conveyor belts used in food processing applications increased by more than 25 times after 5 years of use, including cleaning operations that involved chemical disinfectants and scrubbing of the surface (97). The impact of repeated cleaning processes on coated marble surfaces was simulated by Xi et al. by abrasion, immersion in an acidic solution, and immersion in an alkaline solution (94). They found that the water contact angle decreased after all three simulated cleaning scenarios, indicating the hydrophobicity of coated marble surfaces is impacted by cleaning processes.

Beyond their effect on the surface properties of materials, cleaning methods also have a significant impact on microbial communities present on kitchen surfaces. Cleaning surfaces used to prepare raw poultry with a subsequent rinsing step consistently decontaminated surfaces contaminated with *Campylobacter,* but a significant number of surfaces remained contaminated with low numbers of *Salmonella* (12). A kitchen hygiene model found that cutting boards cleaned by brushing with or without detergent resulted in a nonsignificant decrease in *Escherichia coli* and *Staphylococcus aureus*, but the use of an additional drying or disinfectant step improved the efficacy of cleaning (98). The presence of microbial communities in kitchens and other BE spaces has encouraged the development of antimicrobial cleaning solutions. A test of the efficacy of traditional and antimicrobial cleaning solutions in dishwashers found that antibacterial products significantly reduced aerobic plate count compared to traditional, nonantibacterial products (99). Detergents with acidic or alkaline properties are also commonly used for cleaning surfaces in kitchen settings. Acidic and alkaline detergents have been shown to significantly affect the viability of *Staphylococcus aureus* and *Pseudomonas aeruginosa*, potentially minimizing the spread of contamination (100).

Disinfectants that contain hypochlorite, such as common household bleach, impact the survival of microorganisms on kitchen surfaces and improve the efficacy of cleaning. The use of hypochlorite disinfectant, in addition to detergent and hot water cleaning, resulted in a significant decrease in surfaces contaminated with *Campylobacter* and *Salmonella* compared to the use of only detergent and hot water cleaning (101). Use of disinfectant, with a prescribed protocol, on kitchen surfaces reduced numbers of *Escherichia coli* and *Staphylococcus aureus* when compared to a control group, which continued their regular cleaning procedures (102).

This result indicates that prescribed cleaning protocols may be necessary to achieve greater reductions in bacterial contamination. Targeted use of antimicrobial agents is more likely to reduce the incidence of bacterial contaminants compared to irregular use (103). The use of disinfectants and a prescribed cleaning protocol reduces the risk of microbial contamination and limits microbial regrowth on kitchen surfaces. However, the indiscriminate use of disinfectant can decrease microbial diversity and result in pathogenic species outcompeting other species and becoming dominant (104). In addition to pathogenic competitive advantage, disinfection, particularly below the minimum inhibitory concentration, can result in propagation of ARGs (105, 106).

Lack of cleaning can also impact microorganism survival on kitchen surfaces. *Salmonella* was found to survive for at least a week in food soils drying on a kitchen surface, while *Campylobacter* died rapidly (107). Furthermore, the survival of pathogens was greater in the presence of food residues (cooked rice, whole eggs, and soymilk) compared to the control (108).

### Microbial transfer during cleaning

Cleaning in a kitchen often involves wiping surfaces with cleaning utensils, such as sponges and cloths. However, cleaning utensils themselves can accumulate microorganisms. *Listeria monocytogenes* and fecal coliforms were isolated from wet surfaces, such as dishcloths, and places with stagnant water, such as the kitchen sink, in a study of 250 domestic kitchens (109). In a study of 10 “normal” kitchens in the United States, sponge samples contained large bacterial concentrations, and a high percentage of sponge samples were positive for fecal coliforms (103). Rusin et al. found that the highest concentrations of fecal coliforms, total coliforms, and heterotrophic plate count bacteria were found on sponges/dishcloth samples from 15 household kitchens in Arizona (110).

Cleaning utensils contaminated with microorganisms may be a source for the transfer of microorganisms during use when cleaning and reduce cleaning efficacy. Cloths contaminated with *Salmonella* from the preparation of raw poultry and stored overnight reduce the efficacy of detergent-based cleaning regimes (12). This reduction in efficacy could be attributed to the transfer of *Salmonella* from the contaminated cloths to the surfaces being cleaned. Dishcloths can contain large numbers of microorganisms and easily transfer these microorganisms to other surfaces, acting as reservoirs/disseminators, and effective hygiene procedures should be considered to prevent cross-contamination (111). A laboratory study of bacterial contamination of sponges found that the use of antibacterial dishwashing liquid did not reduce concentrations of bacteria in used sponges, which contained food residues (47). This indicates that repeated use of sponges to clean could aid in the transfer of microorganisms to other surfaces.

Contaminated cleaning utensils can also pose a health risk due to microbial transfer to the user. Cotton-polyester cleaning cloths used in kitchens can pose a significant risk of *Salmonella* infection from hand contamination during the use of these cloths (112). A study in Mexico found that some samples of sponges and dish cloths tested positive for the presence of *Salmonella*, with several isolated serotypes associated with foodborne outbreaks (102). As mentioned in the previous section, the prescribed use of disinfectant can reduce microbial contamination and health risks. Soaking cleaning cloths in hypochlorite after cleaning kitchen surfaces reduces the risk of *Salmonella* infection by almost 100-fold (112). Conversely, disinfectant use can promote transfer of ARGs (105), and these ARB can be transferred between surfaces on utensils that have been cleaned or used for cleaning. Creating an environment that supports a beneficial microbiome, rather than attempting to remove all microbial life, is key to reducing pathogen risk.

## LESSONS LEARNED AND FUTURE DIRECTIONS

The synergy between materials science and microbiology necessitates interdisciplinary expertise and collaboration, making data collection challenging. A review of the literature reveals that the specific material surfaces from which microbiome samples are taken are often unreported. Even when mentioned, materials are typically described in broad terms without detailed characterization. While laboratory studies have demonstrated the impact of material properties on microbial behavior, the physicochemical interactions between substrates and microorganisms, as well as the adhesive properties of microbes, remain largely unexplored in the BE. This lack of information greatly impedes the ability to understand and predict microbial growth on building surfaces. In 2017, the EPA published research agenda “Microbiomes of the Built Environment: A Research Agenda for Indoor Microbiology, Human Health, and Buildings” (3) and listed four priority research areas; the first one is detailed as follows: “Improve understanding of the relationships among building site selection, design, construction, commissioning, operation, and maintenance; building occupants; and the microbial communities found in built environments. Areas for further inquiry include fuller characterization of interactions among indoor microbial communities and materials and chemicals in built environment air, water, and surfaces, along with further studies to elucidate microbial sources, reservoirs, and transport processes.” This highlights the need for better information on the impact of surfaces on MoBEs. Data is required to answer the question of whether surface properties matter.

It must be emphasized that surface properties are not static; surface degradation is possible due to environmental factors, cleaning methods, and the microbiome itself. Cleaning processes, particularly disinfectant use, are proven to reduce the risk of microbial contamination but also impact surface properties. This underscores the need for characterizing the actual surfaces on which microbial sampling occurs, rather than a representative material, since the surface properties may differ from purchased material of the same type.

There are extensive material characterization techniques that can be utilized to provide data on the influence of material properties on the MoBE. Table 2 provides an overview of material properties and characterization techniques.

**TABLE 2:** Overview of material properties and characterization techniques

Material property	Material characterization method	Relevance to MoBE	General summary	Values to report
Chemical composition	Fourier transform infrared spectroscopy (FTIR)/Raman spectroscopy	Surface chemical composition that microbes interact with to attach If microbes attach to branched polymers Chemical composition of mass analyzed	Determines spectral fingerprint and percent matches based on associated libraries	Infrared spectrum Library match
	Nuclear magnetic resonance (NMR)		Determines monomer ratios and branching on polymer structures and protons	Monomer ratios
	Mass spectrometer (MS)		Chemical composition for spectral fingerprint usually paired with another instrument (e.g., ICP-MS)	Spectral fingerprint Library match
Hydrophobicity/hydrophilicity	Contact angle	Ability of material to hold water content	Determines affinity of surface to water	Angle value (°)
Crystallinity and thermal behavior of polymers	Differential scanning calorimetry (DSC) Thermogravimetric analysis (TGA)	Ability of material to resist temperature and sustained cleaning at high temperature	Thermal transitions of material Change in material weight as a function of temperature	Crystallinity T_g_ Graph of temperature vs heat flow
Mechanical properties	Tensile testing Dynamic mechanical analysis (DMA)	Ability to sustain load and mechanical cleaning	Determines many values to describe material strength and ductility	Young's modulus Ultimate tensile strength Yield strength Elongation at break Area under the curve
Surface roughness	Atomic force microscopy (AFM) Optical profilometry	Surface contact for microbes	Determines roughness of surface in relation to smooth comparison	Roughness Z value Qualitative image
Surface imaging	Scanning electron microscopy (SEM)	Imaging surface features and microbes attached to surfaces	Determines surface imaging and qualitative sizes of features	Qualitative images, scale bar quantification
Elemental analysis	X-ray fluorescence (XRF) SEM-EDX (dispersive X-ray spectroscopy)	Elemental effects on microbial survival on surface	Determines elemental composition	Elemental percentages and elemental heat maps onto SEM
Porosity/surface area	X-ray CT (micro and nano)	Ability for microbes to aggregate in pores or create biofilms	Profilometry throughout thickness of material	Porosity images
	Confocal microscopy		Qualitative porosity sizes	Porosity size
	Thermophotometry fluid displacement		Uses freezing/melting of a fluid to determine pore size distribution	Porosity percentage
	Dynamic vapor sorption (DVS)		Absorption of water vapor under controlled humidity	Pore volume

We propose that studies sampling the indoor microbiome should report the materials on which the microbiome was collected, along with material characterization data. Table 3 provides a tiered approach to characterization of material properties relevant to studies focused on the MoBE, allowing for characterization in the field, alongside microbial sampling, as well as techniques that require removal of materials for evaluation in the laboratory using specialized equipment. We believe that the utilization of the material characterization methods shown in Table 3 to better understand microbial attachment, biofilm formation, and longevity of microbial communities on surfaces will generate the data required for a useful predictive framework linking the materials found in the BE and the MoBE. The development of new techniques or instruments to measure surface properties in the field, rather than in the lab, presents an interesting opportunity for further study.

**TABLE 3:** Proposed tiered material characterization for MoBE research

Material property	Tier 0 (method + description)	Tier 1 (method + description)	Tier 2 (method + description)	Example standards	Cost (T0/T1/T2)	Accessibility (T0/T1/T2)	Time (T0/T1/T2)
Surface roughness	Visual inspection − low-magnification defect check	Stylus/optical profilometry − 2D roughness profile (Ra/Rq)	Interferometry/AFM − 3D areal nanoscale map	ISO 21920 25,178	Low/medium/high	Easy/medium/hard	2–10/5–20/10–60 min
Mechanical strength	Manual checks − bend/scratch check	Microhardness/shore-indentation hardness	Tensile/DMA/nanoindent − strength and modulus tests	ASTM D638, E2546, ISO 6507	Low/low–medium/medium–high	Easy/medium/medium–hard	1–5/5–30/10–60+ min
Chemical	Reagent tests − colorimetric indicators	FTIR/Raman/XRF − functional groups and elements	XPS/EDX/MS − surface and bulk chemistry	ASTM E1252, ISO 15472	Low/medium/high	Easy/medium/hard	1–10/1–15/30–120+ min
Hydrophobicity	Droplet visual test − bead/spread behavior	Static contact angle − sessile drop measurements	Adv/Rec + surface energy − dynamic angle and surface energy	ASTM D7334, D7490	Low/low−medium/medium	Easy/medium/medium−hard	1–3/2–10/10–30 min
Water absorbance/porosity	Quick soak − fast mass gain check	Gravimetric absorption − standard % uptake	DVS/porosity − humidity sorption and pores	ASTM D570, ISO 62	Low/low/medium−high	Easy/easy/hard	5–30 min/1–24 h/h-days

Existing methods to measure material properties, such as optical profilometry/AFM (surface roughness), contact angle measurements (hydrophobicity), and FTIR (chemical composition), are relatively accessible and provide useful material property information. If environmental microbiologists are unable to access these methods, collaboration with researchers who have experience with these methods will allow for material metadata to be collected and strengthen our understanding of the role of materials in shaping the MoBE. Additionally, it is imperative for this material data to be integrated with MoBE composition so predictive models can be built, and we can begin identifying best practices for building, operating, and cleaning kitchens. and, by extension, the BE.
